# The School Lunch

**Published:** 1914-05

**Authors:** 


THE SCHOOL LUNCH.—The parent-teachers clubs in many cities and towns are doing a commendable work in providing lunches in the school buildings for the students. These lunches are carefully and scientifically prepared under expert direction and served at cost. The children are encouraged to remain on the school grounds at the noon recess and get their lunches at the school cafeterias. Besides securing better, more healthful and more easily digested and assimilated food, they do not bolt their food hurriedly to get back to the school grounds for a few minutes play during the recess. They can eat deliberately and still have that time for a little recreation so essential to the growing child. At first some parents were disposed to criticize the school lunch movement, because of the expense, but they are learning that the movement is purely philanthropic, the work done without profit, and the food furnished at even a lower cost than the parents can furnish as wholesome food in their own homes. The home noon lunch of the average school boy or girl is often anything but healthful or appetizing, and it is frequently eaten under such conditions that it had better not be eaten at all. But for the organized efforts of the parents and the teachers the movement to furnish school children with appetizing and palatable lunches at school would be a failure, but this co-operation insures the perpetuation of the practice, and will necessarily contribute much to the health of the students.

